# A 5-year retrospective cohort study of denosumab induced medication related osteonecrosis of the jaw in osteoporosis patients

**DOI:** 10.1038/s41598-022-11615-9

**Published:** 2022-05-23

**Authors:** Seoyeon Jung, Jaeyeon Kim, Jin Hoo Park, Ki-Yeol Kim, Hyung Jun Kim, Wonse Park

**Affiliations:** 1 Department of Advanced General Dentistry, College of Dentistry, Yonsei University, 03722 50-1 Yonsei-ro, Seodaemun-gu, Seoul, Korea; 2 Department of Dental Education, College of Dentistry, Yonsei University, 03722 50-1 Yonsei-ro, Seodaemun-gu, Seoul, Korea; 3 Department of Oral and Maxillofacial Surgery, College of Dentistry, Yonsei University, 03722 50-1 Yonsei-ro, Seodaemun-gu, Seoul, Korea; 4 BK21 PLUS Project, College of Dentistry, Yonsei University, 03722 50-1 Yonsei-ro, Seodaemun-gu, Seoul, Korea

**Keywords:** Oral diseases, Endocrinology, Risk factors, Dental treatments, Infection control in dentistry, Oral conditions

## Abstract

Denosumab has been suggested as a first-line therapy for osteoporotic patients. However, a standardized protocol for the prevention of denosumab induced medication-related osteonecrosis of the jaw (MRONJ) has not yet been established. The purpose of this study was to report denosumab induced MRONJ cases, and investigate the factors affecting the occurrence of MRONJ in patients who underwent denosumab and invasive dental treatment (especially tooth extraction) between October 2016 and March 2020. Four of the 98 patients developed MRONJ before and after tooth extraction. The participants were divided into two groups: receiving only denosumab (n = 51) and receiving bisphosphonate as first treatment and denosumab as second treatment (n = 47). There was no significant difference between groups in the occurrence of MRONJ and factors affecting MRONJ. Two out of 4 patients developed MRONJ regardless of invasive treatment after denosumab administration and proceeded with extraction; one patient developed MRONJ after denosumab administration and extraction. The other patient underwent a tooth extraction without osteoporosis treatment, and non-identified MRONJ developed after denosumab administration. MRONJ cases reported in this study show that MRONJ can develop as chronic inflammation without invasive dental treatment; therefore, implementing preventive dental treatment before initiating denosumab treatment is necessary to reduce the occurrence of MRONJ.

## Introduction

Bisphosphonates and the receptor activator of nuclear factor-κB ligand (RANKL) inhibitor denosumab are the most common anti-resorptive agents used in the treatment of osteoporosis^1–3^. Denosumab is suggested as a first-line therapy for postmenopausal osteoporosis patients with high risk of fracture^4,5^. Compared to bisphosphonate’s mechanism of reducing bone resorption by inhibiting differentiation and maturation of osteoclasts and inducing apoptosis^6^, denosumab reduces bone resorption and increases bone strength by inhibiting the differentiation and function of osteoclasts as a single cell antibody against RANKL^7^. However, denosumab must also be evaluated in light of the issues surrounding the use of bone resorption inhibitors, including bisphosphonate. Particularly, medication-related osteonecrosis of the jaw (MRONJ) is one of the most controversial side effects of bone resorption inhibitors^8,9^.

MRONJ is defined based on three criteria: (1) current or previous treatment with antiresorptive or antiangiogenic agents; (2) exposed bone or bone that can be probed through an intraoral or extraoral fistula(e) in the maxillofacial region that has persisted for more than eight weeks; and (3) no history of radiation therapy to the jaws or obvious metastatic disease to the jaws^10^. Since bisphosphonate-related osteonecrosis of the jaw (BRONJ) was first reported in 2003, it continues to be of interest to dental clinicians and researchers^11,12^. However, due to independent reports of denosumab-related bone necrosis, the American Association of Oral and Maxillofacial Surgeons (AAOMS) revised the term BRONJ to MRONJ in 2014^10^.

Various hypotheses have been put forth to explain why bone necrosis occurring after the administration of antiresorptive agents such as bisphosphonate or denosumab is limited to the jaws^13,14^. However, there is insufficient evidence regarding the exact pathogenesis of MRONJ^15^. The most important systemic risk factor for MRONJ is administration of a powerful anti-resorptive agent, such as a nitrogen-containing bisphosphonate or a RANKL inhibitor. For the management of bone metastases in cancer patients, subcutaneous denosumab (120 mg every 4 weeks) was associated with an overall 1.7% risk of MRONJ and an increased risk of developing MRONJ in comparison to high intravenous doses of bisphosphonate (3 mg every 3–4 weeks)^16^. Local risk factors include dentoalveolar surgery (especially tooth extraction), ill-fitting dentures, and existing inflammatory dental diseases (e.g., periodontal disease)^10^.

Previous studies have noted that a 2-month drug holiday before invasive dental procedures should be adequate^17^. However, the AAOMS and American Dental Association (ADA) Council on Scientific Affairs found no evidence that interrupting bisphosphonate and denosumab therapy alters the risk of MRONJ in patients after tooth extraction^10,18^. Accordingly, given the lack of standard duration of the drug holiday for MRONJ prevention in osteoporotic patients with a history of denosumab therapy, there is a need for retrospective clinical cohort studies to develop prevention guidelines and protocols for MRONJ in these patients.

Therefore, we investigated patients diagnosed with osteoporosis who were administered denosumab (Prolia®) at the Department of Endocrinology at Severance Hospital (Seoul, Korea), and underwent invasive dental treatment (tooth extraction) at the Department of Advanced General Dentistry and Oral and Maxillofacial Surgery at Yonsei University Dental Hospital (Seoul, Korea). Accordingly, the aim of this study was to report denosumab induced MRONJ occurrence cases, and the factors affecting the occurrence of MRONJ in patients who underwent denosumab and invasive dental treatment at our institution.

## Material and methods

### Ethics statement

This study protocol was approved by the Institutional Review Board of the Yonsei University Dental Hospital (approval number: 2-2020-0071). Written informed consent was waived, because of the retrospective nature of the study and use of de-identified participant data. This study was performed in accordance with the Declaration of Helsinki.

### Inclusion and exclusion criteria

We screened a total of 159 patients who received denosumab therapy at the Department of Endocrinology at Severance Hospital (Seoul, Korea) and underwent invasive dental treatment at the Department of Advanced General Dentistry and Oral and Maxillofacial Surgery at Yonsei University Dental Hospital (Seoul, Korea) from October 2016 to March 2020.

The inclusion criteria were as follows: age > 20 years, diagnosis of osteoporosis, history of treatment with denosumab (60 mg administered as a single subcutaneous injection once every 6 months) or denosumab combined with bisphosphonate (alendronate, ibandronate, pamidronate, risedronate, or zoledronate), and a history of invasive dental treatment (i.e., tooth extraction) at the Department of Advanced General Dentistry and Oral and Maxillofacial Surgery at Yonsei University Dental Hospital for teeth with hopeless prognosis due to various reasons, including caries, periodontitis, and impaction. The exclusion criteria were as follows: no history of denosumab therapy before and after invasive dental treatment, administration of denosumab 120 mg (Xgeva®) instead of denosumab 60 mg (Prolia®), history of other bone metabolism disorders or administered drugs, secondary osteoporosis due to oncological dose administration in cancer patients, history of denosumab therapy for diseases other than osteoporosis (e.g., hypercalcemia of malignancy, solid cancers, bone metastases, giant cell neoplasm, or multiple myeloma), and a history of denosumab or invasive dental therapy at a different institution, preventing accurate evaluation.

Of the 159 patients (297 teeth) screened, 98 patients (189 teeth) were included in the study and 61 patients (108 teeth) were excluded. The following patients were excluded: 29 patients (53 teeth) who received drugs other than denosumab both immediately before and after invasive dental treatment; 24 patients (43 teeth) who had received other drugs (SERM-raloxifene/bazedoxifene, teriparatide), except denosumab, immediately before invasive dental treatment; 5 patients (9 teeth) who received denosumab treatment or underwent invasive dental therapy at a different institution; 2 patients (2 teeth) with no history of elective tooth extraction (loss of teeth); and 1 patient (1 teeth) who received prophylactic treatment for osteoporosis.

### Variables

Participants’ age, sex, comorbidities, bisphosphonate administration history, bisphosphonate administration period before denosumab administration, number of denosumab dose before extraction, type of drug administered before and after tooth extraction, period from drug cessation to extraction, period from extraction to drug initiation/resumption, reason for extraction, location of extraction (maxillary/mandibular/multiple), and presence or absence of MRONJ were investigated retrospectively.

For patients with MRONJ, location, staging, associated local factors, and the type of MRONJ treatment (conservative/surgical) were additionally investigated. Information on denosumab administration was obtained through prescription records, and electronic medical records were used to obtain information on the invasive dental treatments.

### Statistical analysis

Patient demographics are expressed as n (%) and mean ± standard deviation. The χ^2^ test was used to compare proportions across levels of categorical variables. Due to the low incidence of MRONJ, 40 cases were randomly sampled to obtain stable results. In order to investigate the relationship between MRONJ and comorbidities, multiple logistic regression was used. P-value < 0.05 was considered statistically significant for all analyses. Statistical tests were performed using SPSS statistical software (SPSS for Windows, version 25; SPSS Inc., IBM Corp., Armonk, NY, USA).

## Results

### Baseline characteristics

The baseline characteristics of the 98 participants surveyed in this study, including the average age, sex, and the comorbidities of the patients before tooth extraction are presented in Table 1. The participants’ age ranged from 36 to 91 years, with an average age of 70.5 ± 10.3 years. There were 87 women (88.8%). Of 98 patients diagnosed with osteoporosis, 59 (60.2%) had hypertension, 31 (31.6%) had diabetes, 13 (13.3%) had cancer, and 3 (3.1%) had underlying rheumatoid arthritis.

**Table 1:** Demographics and clinical characteristics.

Characteristics	Study population
Participants, n (%)	98 (100)
Age (years), mean (SD)	70.5 (10.31)
**Sex, n (%)**	
Female	87 (88.8)
Male	11 (11.2)
**Comorbidities, n (%)**	
Hypertension	59 (60.2)
Diabetes	31 (31.6)
Cancer	13 (13.3)
Rheumatoid arthritis	3 (3.1)

Values are n (%), mean (range), as indicated.

The comorbidities has duplicate values.

### Occurrence of MRONJ in Dmab only and BP + Dmab treated patients

The participants were divided into two groups, one receiving only denosumab (Dmab, n = 52 [53.0%]) and another that received bisphosphonate as the first treatment and denosumab as the second treatment (BP + Dmab, n = 46 [46.0%]) (Table 2).

**Table 2:** Characteristics of patients treated with denosumab and bisphosphonate with extraction.

Variables	Overall	Dmab^a^ (n = 52)	BP + Dmab^b^ (n = 46)
Age, mean (SD)	70.47 ± 10.31	69.08 ± 11.82	72.04 ± 8.13
BP administration before Dmab(years), mean (SD)	43.85 ± 47.80		43.85 ± 47.80
Number of Dmab, mean (SD)	2.45 ± 1.63	2.37 ± 1.67	2.50 ± 1.61
Drug cessation to extraction(months), mean (SD)	6.26 ± 4.22	6.22 ± 4.85	6.28 ± 3.86
**Drugs administered after tooth extraction, n (%)**			
Denosumab	53 (54.1)	37 (71.2)	16 (34.8)
Bisphosphonate	4 (4.1)	2 (3.8)	2 (4.3)
Serm	8 (8.2)	2 (3.8)	6 (13.0)
Teriparatide	3 (3.1)		3 (6.5)
None	30 (30.6)	11 (21.2)	19 (41.3)
Extraction to drug initiation/resumption(months), mean (SD)	6.72 ± 8.32	9.46 ± 9.69	2.56 ± 2.03
**Reason for extraction, n (%)**			
Root rest/dental caries/endodontic lesion	52 (53.1)	29 (55.8)	23 (50.0)
Periodontitis	39 (39.8)	19 (36.5)	20 (43.5)
Fracture/crack	12 (12.2)	5 (9.6)	7 (15.2)
Impacted/supernumerary tooth	8 (8.2)	7 (13.5)	1 (2.2)
MRONJ^c^	2 (2.0)		2 (4.3)
**Extraction location, n (%)**			
Maxillary	50 (51.0)	26 (50.0)	24 (52.2)
Mandible	36 (36.7)	18 (34.6)	18 (39.1)
Multiple	12 (12.2)	8 (15.4)	4 (8.7)
**MRONJ/timing with extraction, n (%)**			
Present	4 (4.1)	2 (3.8)	2 (4.3)
Absent	94 (95.9)	50 (96.2)	44 (95.7)

Values are mean (SD), n (%) as indicated.

*BP* bisphosphonate, *Dmab* Denosumab, *SD* Standard Deviation.

^a^Receiving only denosumab.

^b^Receiving bisphosphonate as first treatment and denosumab as second treatment.

^c^MRONJ was induced by existing periodontal inflammation, and as a result, tooth extraction was performed.

The reason for extraction has duplicate values.

In the Dmab only group, the mean age was 69.08 ± 11.82 years. Denosumab was administered on average 2.37 ± 1.67 times before tooth extraction. The mean period from drug cessation to extraction was 6.22 ± 4.85 months. Thirty-seven (71.2%) of the 52 patients received denosumab after tooth extraction, 2 (3.8%) received bisphosphonate, 2 (3.8%) received selective estrogen receptor modulator (SERM), and 11 (21.2%) did not receive any drug. The mean period from tooth extraction to drug initiation or resumption was 7.7 ± 9.1 months. Residual roots, dental caries and other endodontic lesions were the main reasons for tooth extraction, the extraction site was maxillary in 26 patients (50.0%), mandibular in 18 (34.6%), and both in 8 (15.4%).

In BP + Dmab group, the mean age was 72.04 ± 8.13 years which was higher than that in the Dmab group. Bisphosphonate was administered for an average of 43.85 ± 47.80 months before denosumab administration. Denosumab was administered on average 2.50 ± 1.61 times before tooth extraction. The mean period from drug cessation to extraction was 6.28 ± 3.86 months. Sixteen (34.8%) of the 46 patients received denosumab after tooth extraction, 2 (4.3%) received bisphosphonate, 6 (13.0%) received an SERM, 3 (6.5%) received teriparatide, and 19 (41.3%) did not receive any drug. The mean period from tooth extraction to drug initiation or resumption was 2.56 ± 2.03 months. The main reason of tooth extraction was residual root, dental caries, and other endodontic lesions in 23 (50.0%) patients, followed by periodontitis in 20 (43.5%) patients. The extraction site was maxillary in 24 patients (52.2%), mandibular in 18 (39.1%), and both in 4 (8.7%).

Four of the 98 (4.1%) patients developed MRONJ in this study. Two patients belonged to the Dmab group and 2 patients belonged to the BP + Dmab group. Two of 4 patients developed MRONJ before tooth extraction, and 2 patients developed MRONJ after tooth extraction.

### Factors affecting MRONJ development

The factors affecting MRONJ development are presented in Table 3. Among all patients, 43 (43.9%) were in their 70 s, as were 3 (75.0%) patients who developed MRONJ.

**Table 3:** The occurrence of MRONJ according to drug administration and timing.

Variables	Overall	MRONJ (n = 4)	NO MRONJ (n = 94)	*P* value*
**Age, n (%)**				0.71
30–39	1 (1.0)		1 (1.1)	
40–49	1 (1.0)		1 (1.1)	
50–59	16 (16.3)	1 (25.0)	15 (16.0)	
60–69	21 (21.4)		21 (22.3)	
70–79	43 (43.9)	3 (75.0)	40 (42.6)	
80–89	15 (15.3)		15 (16.0)	
90–	1 (1.0)		1 (1.1)	
**Sex, n (%)**				1.00
Female	87 (88.8)	4 (100)	83 (88.3)	
Male	11 (11.2)		11 (11.7)	
**Comorbidities, n (%)**				
Hypertension	59 (60.2)	2 (50.0)	57 (60.6)	0.62
Diabetes	31 (31.6)	2 (50.0)	29 (30.9)	0.30
Cancer	13 (13.3)		13 (13.8)	0.57
Rheumatoid arthritis	3 (3.1)	1 (25.0)	2 (2.1)	0.10
**History of BP use, n (%)**				1.00
Yes	46 (46.9)	2 (50.0)	44 (46.8)	
No	52 (53.1)	2 (50.0)	50 (53.2)	
**Timing of Dmab administration (month)**				
Pre-extraction				0.76
Within 1, n (%)	5 (6.8)	1 (33.3)	4 (5.7)	
1, n (%)	2 (2.7)		2 (2.9)	
2, n (%)	3 (4.1)		3 (4.3)	
3, n (%)	7 (9.6)		7 (10.0)	
4, n (%)	5 (6.8)		5 (7.1)	
5, n (%)	11 (15.1)		11 (15.7)	
6, n (%)	15 (20.5)	1 (33.3)	14 (20.0)	
7–, n (%)	25 (34.2)	1 (33.3)	24 (34.3)	
Post-extraction				0.92
Within 1, n (%)	4 (4.1)		4 (7.5)	
1, n (%)	10 (10.2)		10 (18.9)	
2, n (%)	9 (9.2)		9 (17.0)	
3, n (%)	5 (5.1)		5 (9.4)	
4, n (%)	1 (1.0)		1 (1.9)	
5, n (%)	3 (3.1)		3 (5.7)	
6, n (%)	1 (1.0)		1 (1.9)	
7–, n (%)	20 (20.4)	1 (100)	19 (35.8)	

Values are n (%), as indicated.

The comorbidities has duplicate values.

*Bp* Bisphosphonate, *Dmab* Denosumab, *MRONJ* Medication-related osteonecrosis of the Jaw.

*p-value was obtained from χ^2^ test.

Among 4 patients with MRONJ, 2 had hypertension, 2 had diabetes, and 1 had rheumatoid arthritis (i.e., one had hypertension and rheumatoid arthritis, one had diabetes, and the other had hypertension and diabetes). Two of 46 patients with a history of bisphosphonate administration developed MRONJ, and 2 of 52 patients without a history of bisphosphonate administration developed MRONJ. Among 98 patients, 73 (74.5%) patients administered denosumab before tooth extraction and 53 (54.1%) patients administered denosumab after tooth extraction. One of 3 patients who developed MRONJ had tooth extraction within 1 month after denosumab administration, 1 patient had tooth extraction 6 months later, and 1 patient had tooth extraction after > 6 months. Among the patients who received denosumab after tooth extraction, one developed MRONJ > 6 months after tooth extraction. There was no significant difference between the occurrence of MRONJ and factors affecting MRONJ.

A multiple logistic regression showed no association between the comorbidities of cancer, hypertension, and diabetes and the occurrence of MRONJ (Table 4). However, patients with comorbidities of arthritis were approximately 35 times more likely to occurrence of MRONJ than those without arthritis (P = 0.033).

**Table 4:** Significance of comorbidities by multiple logistic regression.

Comorbidities	OR	95% CI	*P* value
Cancer	0	0	0.999
Arthritis	35.382	1.342–932.515	0.033*
Hypertension	0.328	0.034–3.156	0.334
Diabetes	6.156	0.449–84.341	0.174

*CI* confidence interval, *OR* odds ratio.

*p < 0.05.

### Case description of MRONJ patients

**Figure 1:**
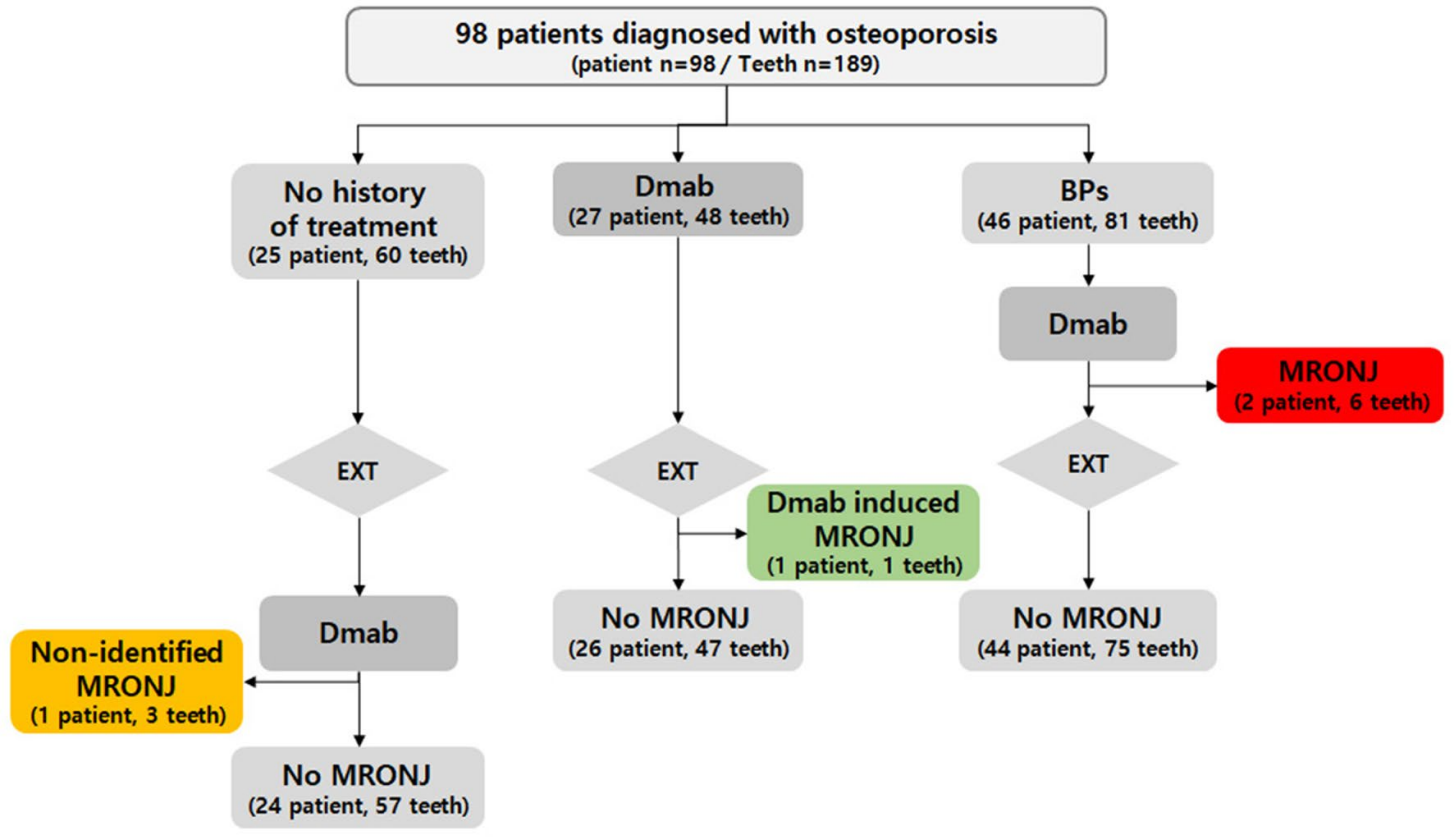
Flow of MRONJ occurrence. Drug administration and MRONJ route before and after tooth extraction in 98 patients diagnosed with osteoporosis. Four patients developed MRONJ. In two of these patients, MRONJ occurred after administration of bisphosphonate and denosumab and before extraction. One patient received denosumab only and developed MRONJ after tooth extraction. The remaining one patient had undergone extraction without prior osteoporosis treatment, and then MRONJ developed after administration of denosumab. *BP* bisphosphonate, *Dmab* denosumab, *EXT* extraction, *MRONJ* medication-related osteonecrosis of the jaw. The dotted line represents the MRONJ flow.

Four patients were diagnosed with osteoporosis in this study population. Following bisphosphonate and denosumab therapy, MRONJ developed before extraction in Cases 1 and 2. In Case 3, after tooth extraction following only denosumab therapy, MRONJ had occurred. In Case 4, the patient had no history of osteoporosis treatment at the time of extraction, and developed MRONJ afterwards, following denosumab administration 1 year after extraction (Fig. 1). Table 5 summarizes the MRONJ clinical characteristics for cases 1–4.

**Table 5:** Case description of MRONJ of osteoporosis patient treated with denosumab.

Patients characteristics			MRONJ characteristics							
Age (years)	Sex	Comorbidities	Previous BP treatment	The period of BP	Number of Dmab before MRONJ	Duration from the last Dmab to the occurance of MRONJ	Local factor	Location	Stage	Conservative/surgical treatment
**Case 1**										
74	Female	Osteoporosis Rheumatoid arthritis Hypercholesterolemia Hypertension	Zoledronate	1 year	3	7 months	Periodontitis	Mn. Rt	2	Extraction surgical debridement
**Case 2**										
76	Female	Osteoporosis Stable angina pectoris Coronary artery disease	Alendronate Ibandronate Zoledronate	7 months	1	6 months	Periodontitis	Mx. Lt	1	Extraction surgical debridement
**Case 3**										
54	Female	Osteoporosis Diabetic mellitus Tracheomalacia			1	5 months	Extraction (extraction 1 month after administration of Dmab)	Mn. Rt	2	Conservative
**Case 4**										
79	Female	Osteoporosis Hypertension Diabetic mellitus Acute myocardial infarction			2	5 months	Non-identified (extraction 1 year before initiating Dmab)	Mn. Ant	2	Conservative

*BP* bisphosphonate, *Dmab* denosumab, *MRONJ* medication-related osteonecrosis of the jaw, *Mn* Mandibular, *Mx* Maxillary, *Rt* Right, *Lt* Left.

#### Case 1

A 74-year-old woman had rheumatoid arthritis, hypercholesterolemia, and hypertension and a history of zoledronic acid (5 mg/100 mg) therapy from March 2015 to March 2016 due to osteoporosis. From October 2017 to October 2018, she received a total of three denosumab doses. In December 2018, there was severe tooth mobility due to localized chronic advanced periodontitis, and extraction was planned to be performed after ≥ 3 to 6 months. Non‐surgical periodontal therapy (i.e., dental scaling and 2% minocycline ointment application [Periocline] into the gingival sulcus) was performed before extraction; however, MRONJ was diagnosed in the right mandibular region 6 months after the last denosumab administration. The drug was changed to Calcitriol [0.25 mcg/Soft Cap], and extraction and sequestrectomy were performed after lesion localization (see Supplementary Fig. S1 online).

#### Case 2

A 76-year-old woman had stable angina pectoris and coronary artery disease, apart from osteoporosis, was treated with endodontics and prosthesis for a crown and root fracture that had occurred approximately 10 years prior. From January 2007 to May 2017, she was administered alendronate and ibandronate, and in August 2017, she was administered zoledronic acid (5 mg/100 mg). One year later, denosumab was administered once. Six months thereafter, MRONJ occurred in the left maxillary region in the presence of localized chronic advanced periodontitis, and bone necrosis was observed up to the mesial root site from the first premolar to the second molar in the radiographic image. Approximately 4 weeks later, tooth extraction and sequestrectomy of the affected area were performed, followed by prosthetic rehabilitation of the missing teeth (see Supplementary Fig. S2 online).

#### Case 3

A 54-year-old woman had diabetes mellitus, osteoporosis. In April 2019, denosumab was administered once. Within a month, the mandibular right second molar had a hopeless prognosis due to a fracture in the tooth, and the tooth was extracted. Three months after extraction, necrotic bone exposure (MRONJ) was found on the lingual side of the extraction site. With denosumab clearing up around October 2019, the area was observed during follow-up and treated conservatively (see Supplementary Fig. S3 online).

#### Case 4

A 79-year-old woman with underlying diseases such as hypertension, diabetes mellitus, and acute myocardial infarction, apart from osteoporosis, did not undergo any osteoporosis treatment before tooth extraction. With the removal of partial dentures used > 10 years, the mandibular anterior teeth had severe mobility and periodontic-endodontic lesions. The teeth were extracted in July 2018. Denosumab was administered twice 11 months after the extraction, and bone loss and increased sclerosis were observed in the anterior mandible in April 2020, 10 months after denosumab administration. Conservative treatment was performed by changing denosumab by raloxifene (60 mg/T) in June 2020 (see Supplementary Fig. S4 online).

## Discussion

Denosumab was reported as an efficacious and favorable benefit–risk profiled osteoporosis medication in the phase 3 randomized trial and open-label extension study. In that study, among the 4550 participants, 13 cases (0.29%) of MRONJ occurred^19^. During the three year FREEDOM trial, in postmenopausal women with osteoporosis, denosumab significantly reduced bone turnover markers, increased bone mineral density, and reduced new vertebral fractures by 68%, along with significant reductions in non-vertebral and hip fractures^20^. Unlike bisphosphonate, the effect of denosumab on bone resorption is immediately reduced after treatment discontinuation, because it is not resorbed by the bone. Thus, physicians should pay attention to the duration of denosumab administration, and if denosumab is discontinued, it must be changed to another anti-resorptive agent to prevent the increased risk of vertebral fractures^20^.

Various factors have been implicated as causes of MRONJ, but recently, it was suggested that anti-resorptive agents affect the immune function of the bone. Tooth extraction has been reported as a predisposing factor of MRONJ in approximately 45–61% cases, but the prevalence and incidence rates of other spontaneous occurrences, which are the second largest factor and present as periodontal or periapical lesions, implants, or dentures, as well as extractions, have not been reported^21,22^. In animal studies, lesions similar to MRONJ have been reported in cases of periapical inflammation and periodontitis^10,23^. However, there is still a lack of clinical research on denosumab-induced MRONJ and its incidence, and no protocol has been established yet for invasive dental treatment of the patients receiving this drug.

In this study, we reported four cases of MRONJ in patients using denosumab, out of 98 patients who received either only denosumab injections or with bisphosphonate and who underwent tooth extractions. Although all four cases have something in common, they are cases of MRONJ occurring after denosumab administration, each situation is distinct. Two of the four MRONJ cases (Cases 1–2) had a history of bisphosphonate treatment. As for predisposing local factors of MRONJ occurrence, Case 1 and 2 are due to worsening of the chronic inflammation of the existing lesion after the denosumab administration, Case 3 was due to tooth extraction, and Case 4 as a non-identified occurrence. This means that chronic active periodontitis may have been a significant risk factor in the two cases, suggesting the importance of preventive dental treatment. Marx et al. reported that advanced periodontitis was the cause of bone exposure in 28.6%^24^, and existing inflammatory lesion such as periodontal and apical disease are known risk factors^25^, but that for Dmab-induced MRONJ has not yet been reported.

Several hypotheses have been raised regarding the occurrence of MRONJ in sites of chronic inflammatory lesions, and an M1 macrophage shift is considered the most likely explanation^26,27^. In previous studies, it was reported that osteonecrosis occurred when anti-resorptive agents were administered to animal models that induced pulpal and periodontal inflammation^28–31^. Cheong et al. observed an increase in bisphosphonate uptake at the apex in mice with periapical disease, and suggested that this may be associated with ONJ development^32^. Recently, Kim et al. reported that the incidence of osteonecrosis decreases if pre-inflammatory conditions are removed prior to extraction and emphasized the importance of pulpal and periodontal disease as local factors in the occurrence of MRONJ^23^. Osteonecrosis occurs when bisphosphonate or RANKL inhibitors are administered in the presence of inflamed pulpal or periodontal tissue, because anti-resorptive agents affect the function of various immune cells, such as of neutrophils and polymorphonuclear leukocytes, macrophages, and dendritic cells^33–38^.

The most recently studied immune cells are macrophages, and a particular focus on macrophage polarization. Polarization refers to the different ratio of M1 macrophages having pro-inflammatory and antimicrobial properties and M2 macrophages having anti-inflammatory properties^39,40^. Zhang et al. reported that IL-17 mediated M1/M2 macrophage alteration is related to the development of BRONJ^35^, and Tamaki et al. reported that dynamic M1/M2 macrophage polarization was induced by the anti-RANKL antibody in a MRONJ model^41^. In this context, Hoefert et al. reported that patients with MRONJ had a compromised macrophage function when compared to patients with osteoradionecrosis and osteomyelitis^37^, and Paschalidi et al. found M1/M2 macrophage polarization in macrophages of patient tissues in MRONJ, and that macrophages shift to the M1 phenotype at stages 2 and 3, as compared to stage 1 of MRONJ^26^.

In Case 3, the extraction was performed one month after the administration of a single denosumab dose injection. MRONJ developed four months after surgery. The lesion was classified as grade 2 MRONJ, and cured with a five-month conservative treatment. This case suggests a one month time between the denosumab injection and extraction may be too short.

The patient in Case 4 had no history of bisphosphonate administration, and the development of MRONJ was “non-identified” after two administrations of denosumab a year after extraction. The most common causes of MRONJ are tooth extraction and obvious periodontal disease, followed by “non-identified” occurrences with no identifiable cause^24^. Non-identified occurrence is the third most common group of MRONJ cases reported in the literature, with an estimated prevalence between 16 and 70%^42^. Most of these non-identified occurrences were found to occur in relation to the lower posterior tooth region, but the lesion described in Case 4 developed anteriorly. In this case, the teeth with severe mobility and periodontic-endodontic lesion had been extracted. Although the lesion was completely removed during the operation, MRONJ occurred at the surgical site 21 months after extraction. Therefore, we classified this case as a non-identified occurred MRONJ, not an occurrence in an existing lesion. Importantly, a non-identified MRONJ occurrence poses the risk of a pathological fracture of the jaw after denosumab administration.

In this study, the incidence of MRONJ after denosumab administration was 4.1% and no statistically significant factor was found that influenced the incidence of MRONJ in patients receiving denosumab treatment. To prevent MRONJ, which degrades the patient's quality of life, both the risk of fracture and osteonecrosis of the jaw risk should be considered. In case of the risk of fracture and ONJ are high but invasive dental treatment is required, the discontinuation of denosumab should be considered. However, denosumab increases fracture risk upon discontinuation of treatment, so teriparatide can be prescribed if not contraindicated or alternative osteoporosis drugs should be prescribed during discontinuation of treatment, and monitoring is required.

This study provides information on the incidence of MRONJ in patients with denosumab but has limitations. First, it was a retrospective study using data from a single institution. In addition, bone turnover markers (BTM) were not investigated because all participants' BTMs were not measured at the same time. However, clinical data only makes it difficult to predict the occurrence of MRONJ and requires another predictive factor, therefore molecular biomarkers need to be included in future research.

## Conclusion

Because it is difficult to predict MRONJ occurrence in patients under denosumab therapy, oral healthcare before, during, and after denosumab treatment is essential. Before the initiation of denosumab therapy, it is necessary to control existing inflammatory lesions through the preventive dental care. During denosumab therapy, it is important for both physicians and dentists to have common consensus of drug changes according to the risk of fracture and osteonecrosis when performing invasive dental treatment. In addition, continuous periodic dental management needs to be performed during denosumab treatment to prevent “non-identified” MRONJ. In the future, further multi-center or molecular biological studies are needed.

## Supplementary Information

Supplementary Figures.

## Data Availability

The datasets generated during and/or analysed during the current study are available from the corresponding author on reasonable request.
