# Supplementary material for: A 5-year retrospective cohort study of denosumab induced medication related osteonecrosis of the jaw in osteoporosis patients
Source: Sci Rep. 2022 May 23;12:8641. doi: 10.1038/s41598-022-11615-9 (PMC9126865; doi:10.1038/s41598-022-11615-9)
Supplement: Supplementary file 1 — Supplementary Figures. [file 41598_2022_11615_MOESM1_ESM.pdf]

Supplementary Figure 1

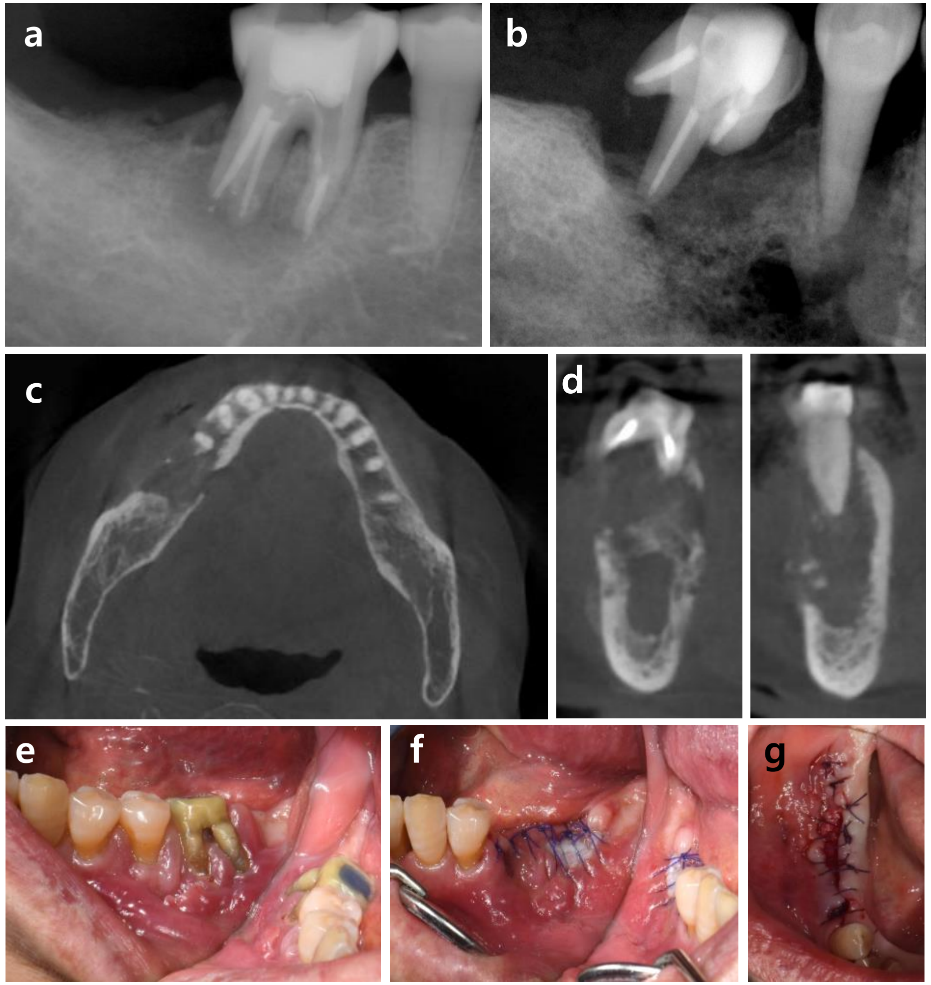

Supplementary Figure 1. Case 1. (**a**) A 74-year-old woman with a history of zoledronic acid and denosumab therapy, who underwent root canal treatment due to fistula formation in relation to the right mandibular first molar, and proceeded with conservative treatment due to chronic advanced periodontitis before extraction. (**b**) MRONJ occurred 6 months after denosumab cessation. (**c**, **d**) Bone loss and cortication in the upper mandibular canal and bone necrosis inside the lesion before tooth extraction. (**e**) An image of the MRONJ lesion in the right mandibular region. (**f**) Tooth extraction and sequestrum removal. (**g**) Second sequestrectomy.

Supplementary Figure 2

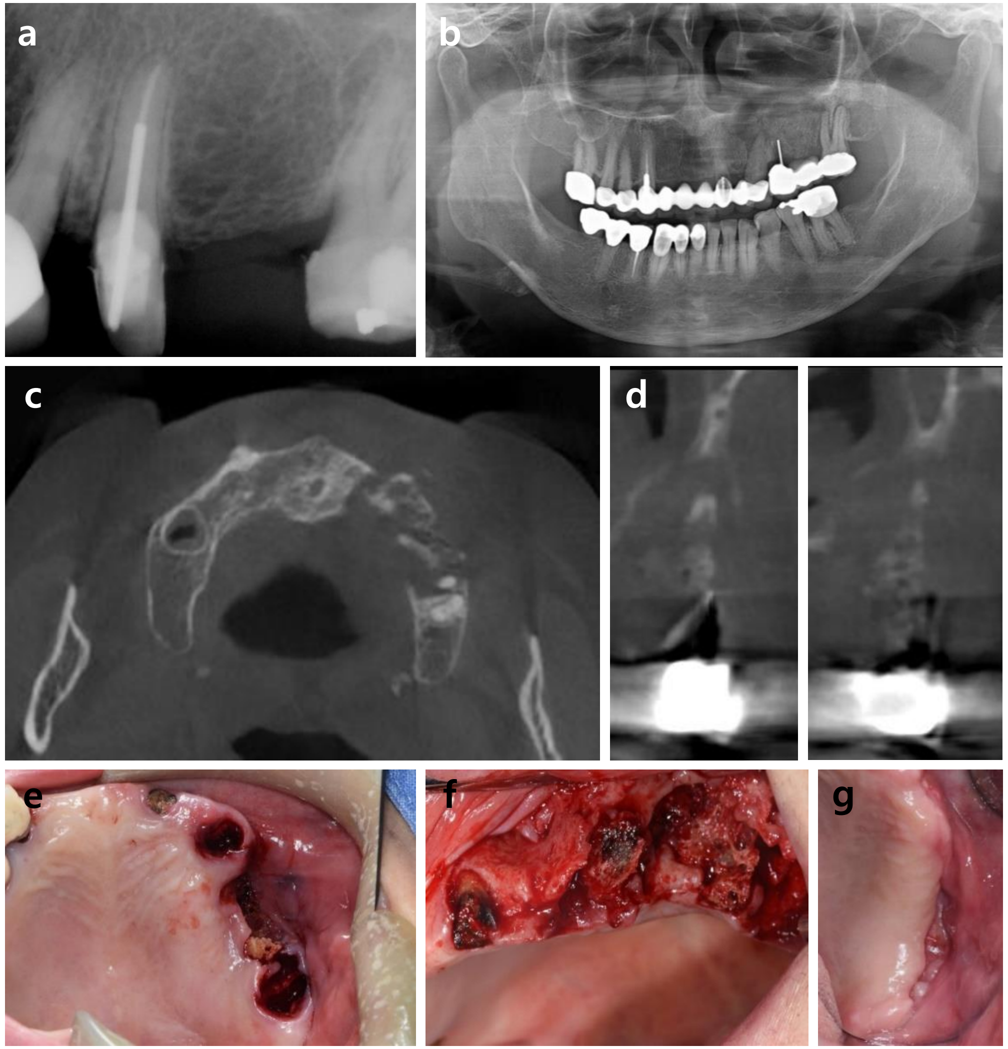

Supplementary Figure 2. Case 2. (**a**, **b**) A 76-year-old woman who was using BP for >3 years and had undergone endodontic and prosthetic surgery without extraction for the treatment of a crown and root fracture of the left maxillary second premolar. (**c**, **d**) Radiography of the MRONJ lesion, with necrotic bone on the mesial aspect of the root on the left maxillary region. (**e, f**) An image acquired immediately after performing extraction and sequestrectomy from the first premolar to the second molar. (**g**) Although the gingiva was swelling, healing process was observed one month after dental surgery.

Supplementary Figure 3

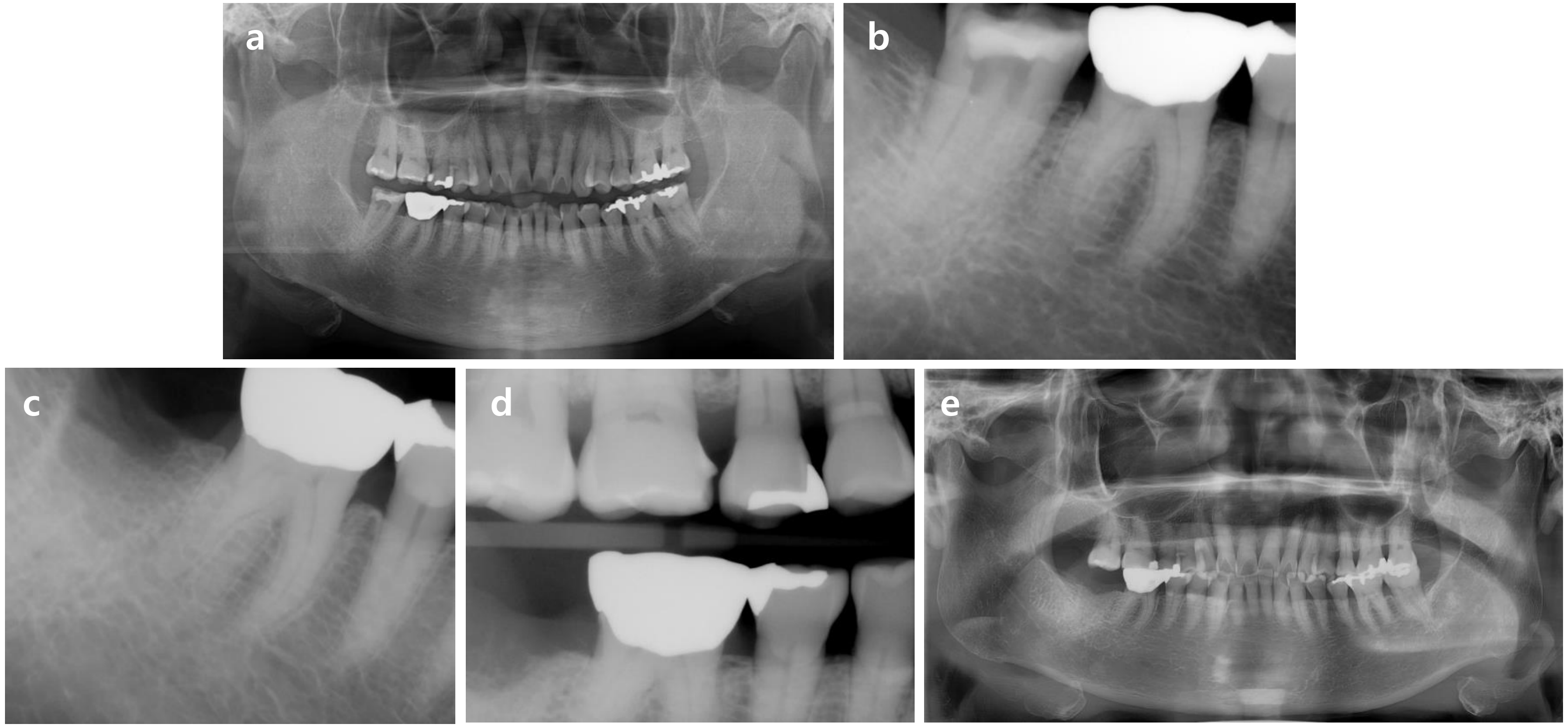

Supplementary Figure 3. Case 3. (**a**, **b**) A 54-year-old woman who was administered denosumab and required dental extraction due to a fracture in relation to the right mandibular second molar with hopeless prognosis. (**c**) Radiography performed immediately after tooth extraction. (**d**) MRONJ occurred on the lingual side of the right mandibular region 3 months after extraction. (**e**) Healing state after 5 months of conservative treatment.

Supplementary Figure 4

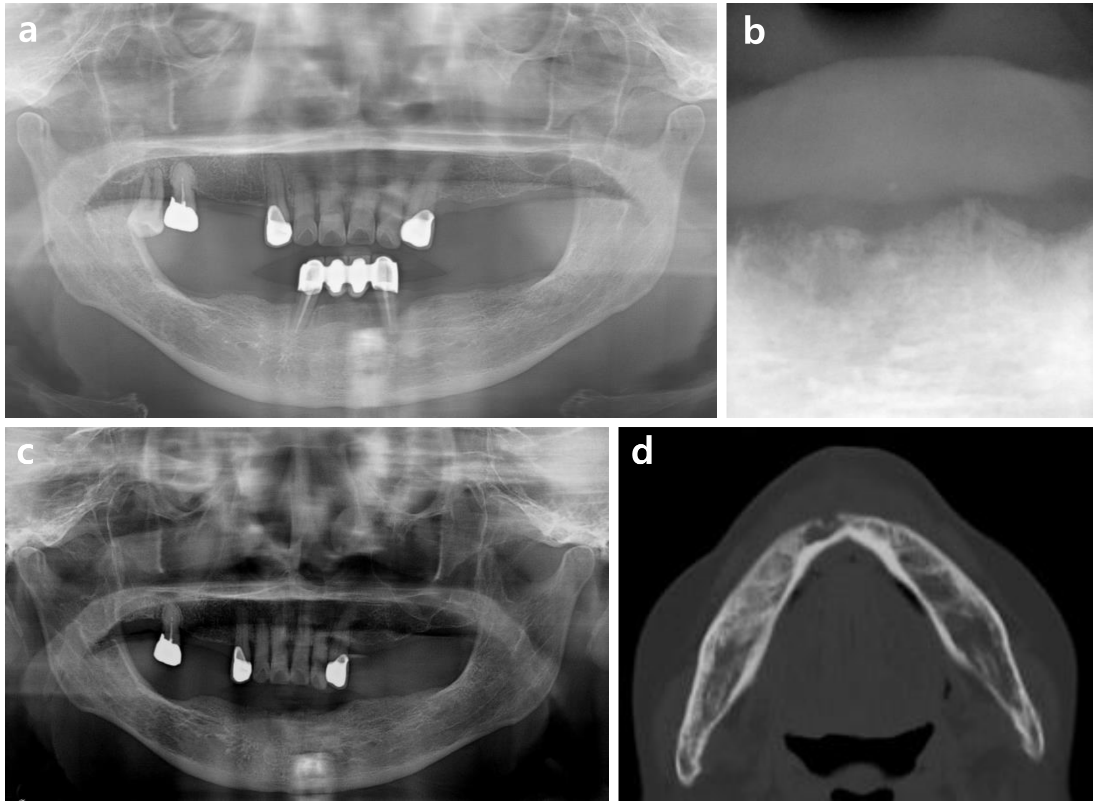

Supplementary Fig. S4 Case 4. (**a**) A 79-year-old woman with no history of osteoporosis treatment required dental extractions due to periodontic-endodontic lesions of both mandibular lateral incisors. (**b**, **c**) Three months after the extractions, there was bone loss and fistula formation on the edentulous ridge. (**d**) Diffuse bone loss was observed in relation to the mandibular anterior teeth and significant sclerosis was observed around the area.
